# Integrated data mining and network pharmacology to explore the prescription patterns from a senior TCM oncologist’s clinical practice in treating chemotherapy-induced hand-foot syndrome

**DOI:** 10.1097/MD.0000000000044872

**Published:** 2025-10-03

**Authors:** Li Deng, Hongjing Chen, Gang Xie

**Affiliations:** a College of Integration of Traditional Chinese Medicine and Western Medicine, Southwest Medical University, Luzhou, China; b Wenzhou Graduate Joint Training Base, Zhejiang Chinese Medical University, Wenzhou, China; c Institute of Traditional Chinese Medicine of Sichuan Academy of Chinese Medicine Sciences (Sichuan Second Hospital of T.C.M), Chengdu, China.

**Keywords:** data mining, hand-foot syndrome after chemotherapy, medication laws, network pharmacology, traditional Chinese medicine

## Abstract

Hand-foot syndrome (HFS) is a common and refractory adverse effect of chemotherapy lacking specific therapeutic strategies currently. Traditional Chinese medicine (TCM) has shown empirical efficacy in clinical HFS management. This study integrated data mining and network pharmacology to systematically elucidate the medication principles and molecular mechanisms underlying Professor Gang Xie’s prescriptions for HFS. All medical records from Professor Xie’s specialist clinic (January 2020 to March 2025) were retrospectively collected and standardized in Excel. Prescriptions were analyzed through frequency statistics, association and clustering. Active ingredients of core herb pairs and their disease-related targets were identified using TCMSP, HERB, GeneCards, PharmGKB and GEO databases. Protein–protein interaction (PPI) networks, gene ontology (GO), and Kyoto encyclopedia of genes and genomes (KEGG) pathway analyses were performed. Molecular docking validated interactions between key bioactive compounds and targets. This study involved 217 prescriptions containing 150 herbs. Core herb combinations comprised *Radix Astragali* (Huangqi), *Poria* (Fuling), and *Radix Pseudostellariae* (Taizishen), predominantly classified as spleen-tonifying agents with warm properties, targeting lung, spleen, and stomach meridians. Network analysis identified 67 bioactive compounds and 899 disease targets. Quercetin, kaempferol, acacetin and luteolin were identified the key ingredients. The core targets (TP53, STAT3, PIK3CA, HSP90AA1, AKT1, CTNNB1, PI3KR1, MAPK1) were enriched in MAPK and PI3K-Akt signaling pathways. Molecular docking confirmed strong binding affinity between key compounds and targets. Professor Xie’s therapeutic strategy for HFS emphasizes “spleen fortification, phlegm elimination, and stasis resolution.” The core herb combination likely exerts anti-HFS effects via modulation of MAPK and PI3K-Akt pathways, providing a pharmacological basis for TCM-driven HFS management.

## 1. Introduction

Cancer has become an important factor in threatening human survival.^[1]^ According to statistics, there were about 20 million new cases of cancer in the world in 2022, and about 9.7 million people died of cancer every year.^[2]^ Chemotherapy is one of the main means in treating malignant tumors, which plays an important role in killing tumor cells, prolonging survival time.^[3–6]^ However, longterm application of chemotherapeutic agents can also induce many adverse effects, and the chemotherapy-induced peripheral neuropathy (CIPN) is the common adverse effects of chemotherapeutic agents such as platinum and paclitaxel.^[7,8]^ Approximately 50% of patients with chemotherapy would develop hand-foot syndrome after chemotherapy (HFS), which is manifested by numbness, tingling and pain, even complicated by edema, cracking and hyperpigmentation in hands and feet.^[9–13]^ HFS not only reduces the quality of life, but also may cause some patients to reduce the dose of chemotherapeutic agents, or even interrupt chemotherapy, thus affecting the therapeutic efficacy of anti-tumor procedure.^[14,15]^ Current medicine mainly relies on topical steroids and analgesics for HFS. But these treatments may cause side effects and induce addiction, meanwhile, there is a lack of effective intervention for sensory abnormalities such as numbness.^[16–18]^

As an experiential medicine, traditional Chinese medicine (TCM) has a long history of several thousand years and was widely practiced in multisystemic diseases, including gastritis, pulmonary fibrosis and osteoporosis.^[19–21]^ Based on the convergence of traditional experience and modern technology, TCM exhibited an excellent clinical potential. For example, inspired by ancient TCM literature, the extraction of artemisinin has saved millions of lives from malaria.^[22]^ In current studies, employment of Chinese herbal formulas in cancer treatment directly or indirectly has been a novel field.^[23–29]^ Chinese medicine believes that HFS can be classified as “bi syndrome,” and its pathogenesis is mainly related to qi deficiency and blood stasis pattern. Scientific evidences have shown that TCM formulas can effectively ameliorate the HFS and improve chemotherapy adherence.^[30–36]^ Therefore, identifying these effective medications from famous TCM practitioner can be helpful for future clinical and scientific practice.^[37–40]^

**Figure 1.**
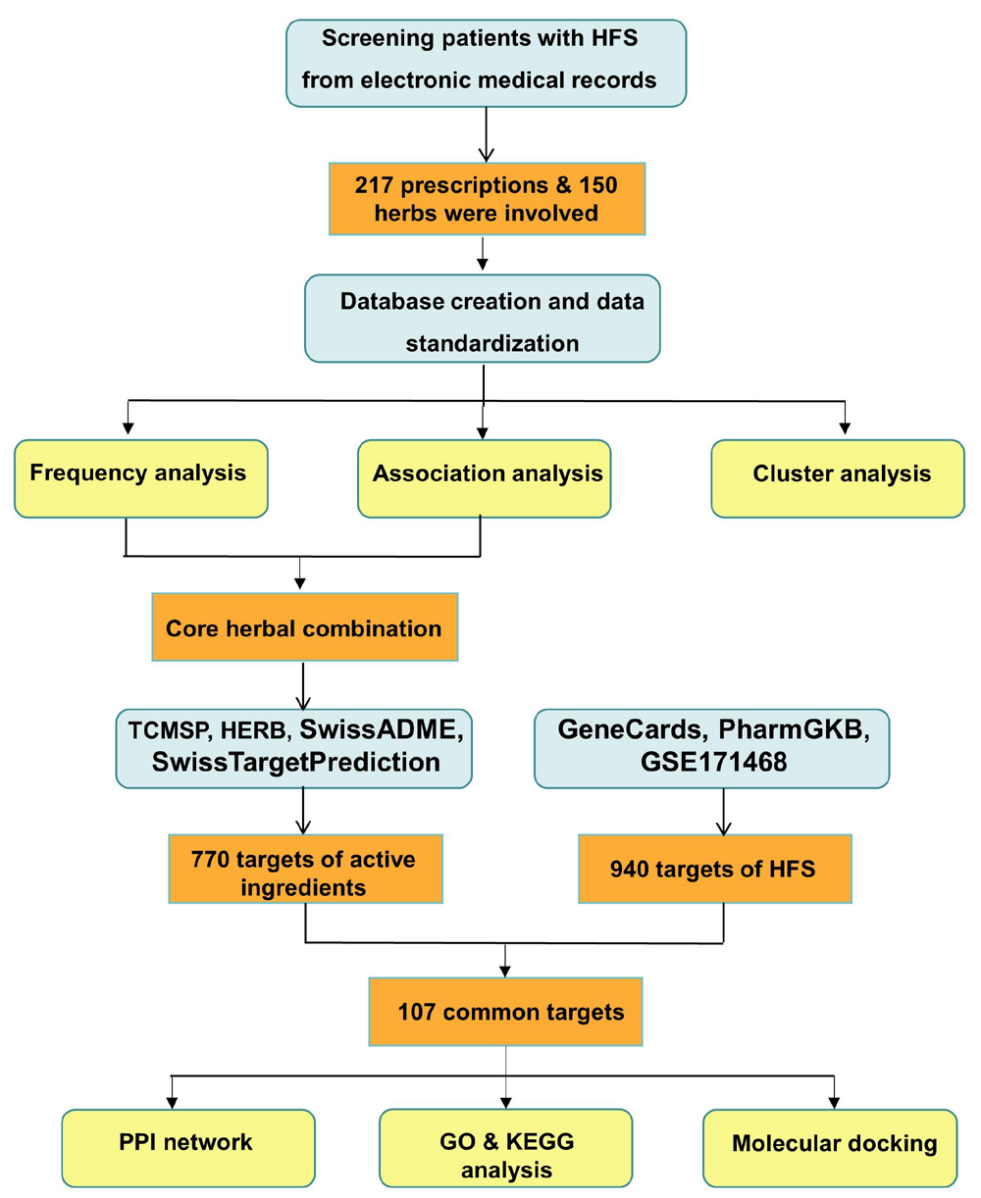
The flowchart of this work.

Professor Gang Xie, the reputable Chinese medicine practitioner of Sichuan Province, has deeply engaged in the field of medical practice of TCM to prevent and treat malignant tumors for decades. Professor Gang Xie is proficient in the application of Chinese medicine to reduce the postoperative complications of malignant tumors and the toxicity of radiotherapy and chemotherapy. Prescription data mining and network pharmacology are the methods to analyze current effective prescriptions and their mechanisms to guide future clinical research.^[37,41–43]^ This study aimed to analyze the medication pattern of Professor Gang Xie in the treatment of HFS and further explored the potential mechanisms, thereby providing data support and theoretical reference for future investigation in HFS (Fig. 1).

## 2. Materials and methods

### 2.1. Data source

The prescription data used in this study were obtained from the outpatient medical records of TCM Workshop of Professor Gang Xie, which contained chief complaint, present illness history, past medical history, prescriptions and other necessary information. This study was approved by the Ethics Committee of Sichuan Second Hospital of T.C.M and waived the requirement for informed consent because of anonymized data (2025ZX-22).

### 2.2. Prescriptions selection

Cases were selected from patients with malignant tumors who have been treated from January, 2020 to March, 2025 in the outpatient clinic of Professor Xie Gang, Department of Oncology, Sichuan second hospital of TCM. Criteria for inclusion: complete information of medical records (present illness history, past medical history, prescription information, etc); the medical records clearly recorded that the patients had the syndromes of HFS (numbness or pain); and the medical records recorded that the syndromes were relieved or disappeared after taking TCMs, then the previous prescription was incorporated. Following were the exclusion criteria: previous medical history records that patients combined with other diseases that can cause paresthesia of hands and feet, such as cervical spondylosis, diabetes etc; and those who have mental or intellectual disorders so that they were unable to accurately express the sensation.

### 2.3. Data entry standardization

The prescription data were imported into the Excel table and standardized with reference to the “Pharmacopoeia of the People’s Republic of China”^[44]^ and “WHO international standard terminologies on TCM,”^[45]^ including the name, flavors and natures of drugs. For example, stir-frying Huangqi is standardized as Huangqi, 4 natures of drugs were unified as “cold, hot, warm, cool and neutral properties,” the 5 flavors were unified as “sour, bitter, sweet, salty, pungent, bland and astringent.” Finally, the duplicated prescriptions were removed. This procedure was checked and confirmed by the second researcher to validate the data.

### 2.4. Data analysis

Microsoft Excel 2019 was utilized to summary the frequency, function category, natures, flavors and meridians of Chinese medicines. IBM SPSS Modeler 18.0 was used to carry out correlation analysis of these herbs with Apriori algorithm, with setting the degree of support  ≥ 10% and the confidence level ≥ 90%. IBM SPSS Statistics 27 was used to perform systematic cluster analysis of high-frequency herbs, with the method of intergroup linkage and the measurement criterion of Pearson correlation. Finally, the above findings were visualized in Origin 2024 and Cytoscape 3.8.0.

### 2.5. Screening of active ingredients drugs and potential targets for treating disease

The active ingredients of Chinese medicines were obtained from the TCMSP (https://tcmspe.com/)^[46]^ and HERB^[47]^ databases. The inclusion criteria in TCMSP were oral bioavailability ≥ 30% and drug-likeness ≥ 0.18, and the ingredient-related targets were derived in this database. The components obtained in the HERB were submitted to the SwissADME platform (http://www.swissadme.ch/) and the screening conditions were set as follows: GI-absorption was “High,” and at least 3 items of Druglikeness are “Yes.” The screened ingredients were subjected to target prediction on SwissTargetPrediction platform (http://www.swisstargetprediction.ch), and the targets with confidence level of 0 were removed. The above components and targets are subjected to redundancy removal management.

Disease related targets were obtained from GeneCards (https://www.genecards.org/), PharmGKB (https://www.pharmgkb.org/) and GEO (www.ncbi.nlm.nih.gov/geo/) datasets, the keyword “Chemotherapy induced hand-foot syndrome” was designated as the search term. The duplicate targets were eliminated and the collected targets were intersected with the targets of active ingredients. The intersecting targets were visualized using the Venny 2.1.0 platform (https://bioinfogp.cnb.csic.es/tools/venny/).

### 2.6. PPI network analysis

The intersecting targets were imported into String platform (https://string-db.org/), and the species was set as “Homo sapiens” with a confidence level ≥ 0.9. Then the protein–protein interaction (PPI) network was constructed in Cytoscape 3.8.0 software.

### 2.7. GO and KEGG enrichment analysis of intersection targets

R4.1.1 was used to perform gene ontology (GO) and Kyoto encyclopedia of genes and genomes (KEGG) enrichment analysis of intersecting targets. The results of GO and KEGG enrichment analyses were imported into the Weishengxin platform (http://www.bioinformatics.com.cn/) for visualization.

### 2.8. Construct ingredients-intersection targets network

The active ingredients and intersecting targets of drugs were imported into Cytoscape 3.8.0 to construct the network diagram, and the core active ingredients were obtained through degree value screening.

### 2.9. Molecular docking

The 2D chemical structure of the core active ingredients were downloaded from the PubChem database (https://pubchem.ncbi.nlm.nih.gov/) and converted into 3D chemical structure using Chem3D software, then the structure was saved as the ligand file in mol2 format. The PDB files of targets were obtained from the PDB database (https://www.rcsb.org/). After dehydrogenation, solvent and organic molecules removal in Pymol 2.6 and AutoDockTools 1.5.6, the receptor proteins were docked to the compounds and the minimum binding energy was calculated in AutodockVina 1.1.2. Finally, R4.1.1 was applied to visualize the docking energy.

## 3. Results

### 3.1. Frequency analysis

**Figure 2.**
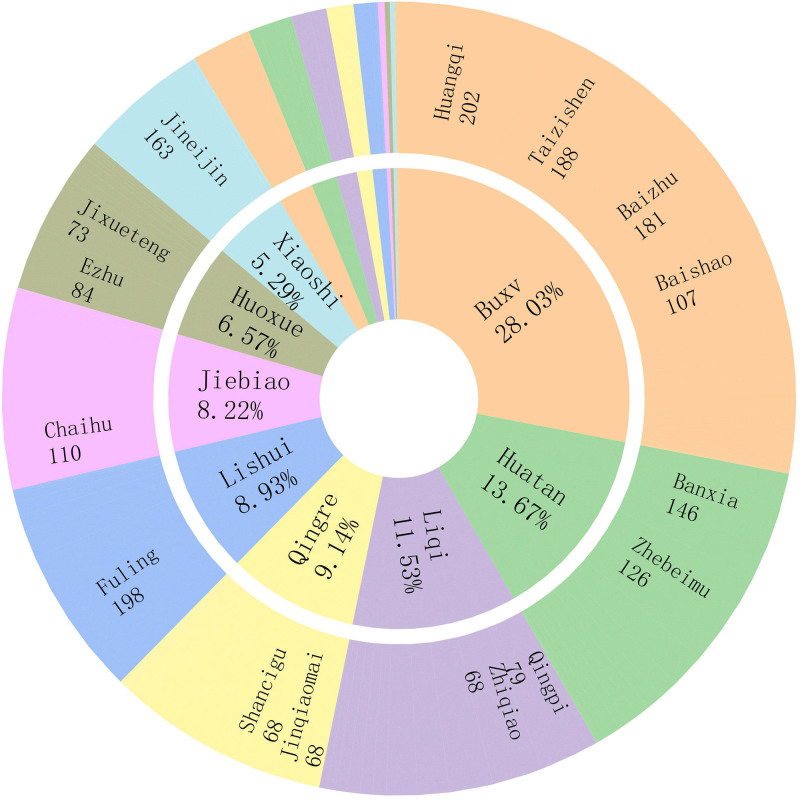
Primary functional categories of TCMs and their associated high-frequency herbs. The inner ring displays each primary functional category along with its frequency rate (e.g., Buxv for tonic medicines), while the outer ring presents the frequency counts of the corresponding high-frequency herbs. TCM = traditional Chinese medicine.

**Figure 3.**
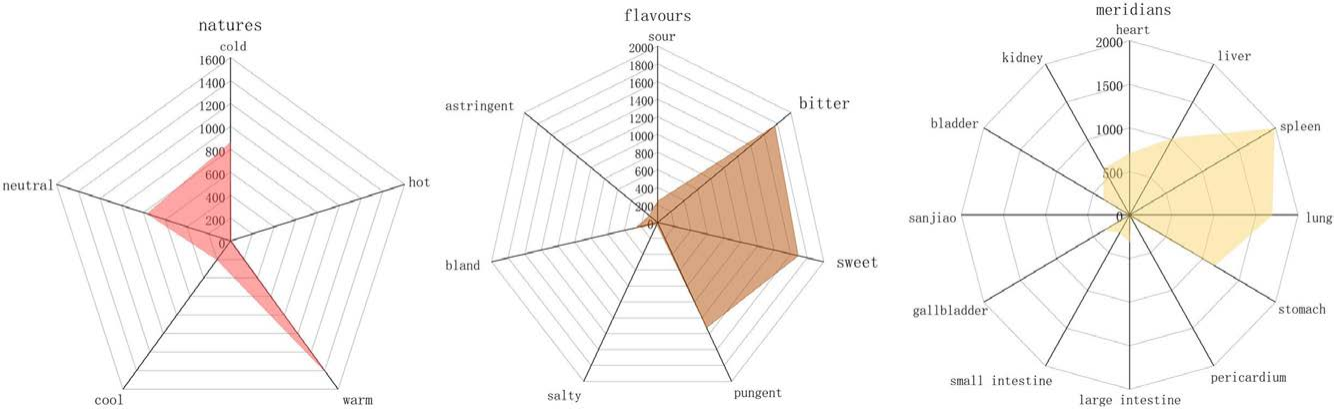
The frequency distribution of medicines in natures, flavors and meridians.

A total of 41 cases were included in this study, and after removing the duplicates, a total of 217 prescriptions which involving 150 medicines. The number of herbs in each prescription was from 12 to 18. The total herbal application frequency was 3254, with 22 high-frequency herbs (frequency ≥ 40), among which Huangqi was the highest, followed by Fuling, Taizishen, *Rhizoma, Atractylodis macrocephalae* (Baizhu) and *Gallus gallus domesticus* (Jineijin) (Table 1).The 150 medicines were categorized into 17 groups according to their functions, among which tonic medicines (Buxv, n = 917), phlegm-transforming medicines (Huatan, n = 447), qi-regulating medicines (Liqi, n = 377), heat-clearing medicines (Qingre, n = 299), urination-promoting medicines (Lishui, n = 292), exterior-releasing medicines (Jiebiao, n = 269), blood-circulating medicines (Huoxue, n = 215), digestion-promoting medicines (Xiaoshi, n = 173) were the predominant types (Fig. 2). Frequency statistics revealed that medicines with the nature of warm, and flavors of sweet and bitter were commonly prescribed. And most of them belonged to the meridians of spleen, lung and stomach (Fig. 3).

**Table 1:** The high-frequency herbs (≥40).

Sequence	Herbs	Frequency	Frequency rate	Sequence	Herbs	Frequency	Frequency rate
1	*Radix Astragali*	202	0.9309	12	*Spatholobi caulis* (Jixueteng)	73	0.3364
2	*Poria*	198	0.9124	13	*Rhizoma Fagopyri Cymosi* (Jinqiaomai)	68	0.3134
3	*Radix Pseudostellariae*	188	0.8664	14	*Pseudobulbus Cremastrae seu Pleiones* (Shancigu)	68	0.3134
4	*Rhizoma Atractylodis macrocephalae*	181	0.8341	15	*Fructus Aurantii* (Zhiqiao)	68	0.3134
5	*Gallus gallus domesticus*	163	0.7512	16	*Fructus Amomi* (Sharen)	61	0.2811
6	*Rhizoma Pinelliae* (Bnxia)	146	0.6728	17	*Vladimiria souliei* (Chuanmuxiang)	58	0.2673
7	*Bulbus Fritillariae thunbergii* (Zhebeimu)	126	0.5806	18	*Radix Angelicae sinensis* (Danggui)	49	0.2258
8	*Radix Paeoniae Alba* (Chaihu)	110	0.5069	19	*Ramulus Cinmomi* (Guizhi)	46	0.2120
9	*Radix Paeoniae Alba* (Baishao)	107	0.4931	20	*Trichosanthes Kirilowii* (Gualou)	45	0.2074
10	*Rhizoma Curcumae* (Ezhu)	84	0.3871	21	*Bulbus Allii Macrostemi* (Xixebai)	42	0.1935
11	*Pericarpium Citri Reticulatae Viride* (Qingpi)	79	0.3641	22	*Pericarpium Citri Reticulatae* (Chenpi)	41	0.1889

### 3.2. Association analysis

**Figure 4.**
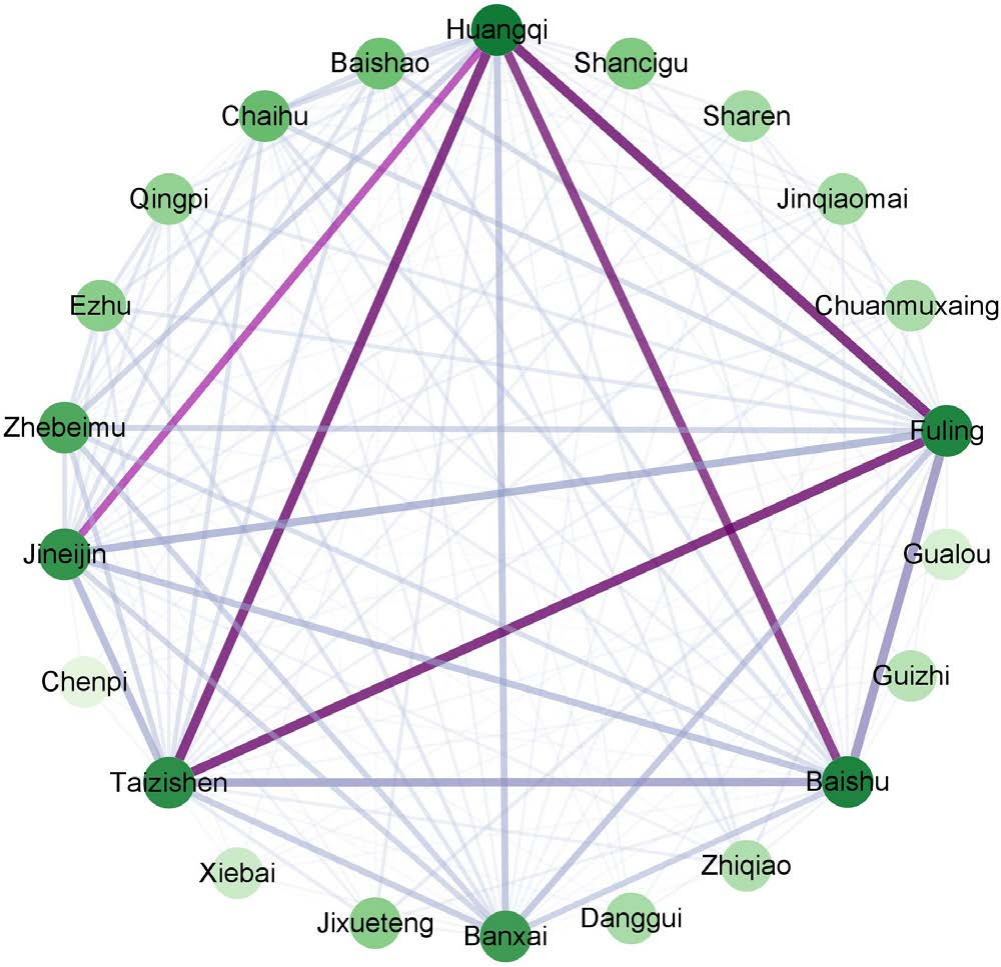
The medicine association network of high-frequency herbs.

All the prescription data were analyzed for associations using IBM SPSS Modeler 18.0. The data were ranked by support values and 20 pairs of high-frequency herbs with a support rate ≥ 70% were identified. Notably, the pair of Taizishen and Huangqi exhibited the highest support and the combination of Taizishen, Fuling and Huangqi emerged as the optimal 3-item association. The top 20 association combinations were list in Table 2. Finally, the network with the highest association strength, primarily composed of high-frequency herbs, was visualized using Cytoscape 3.8.0 (Fig. 4).

**Table 2:** Medicines association rule (top 20).

Sequence	Medicine associations	Frequency	Support	Confidence	Lift
1	Taizishen–Huangqi	200	0.9217	0.9150	1.06
2	Fuling–Huangqi	200	0.9217	0.9250	1.01
3	Taizishen–Fuling	198	0.9124	0.9242	1.07
4	Huangqi–Fuling	198	0.9124	0.9343	1.01
5	Fuling–Taizishen	188	0.8664	0.9734	1.07
6	Huangqi–Taizishen	188	0.8664	0.9734	1.06
7	Tizishen–Fuling and Huangqi	185	0.8525	0.9622	1.11
8	Huangqi–Taizishen and Fuling	183	0.8433	0.9727	1.06
9	Fuling–Taizishen and Huangqi	183	0.8433	0.9727	1.07
10	Taizishen–Baizhu	181	0.8341	0.9116	1.05
11	Fuling–Baizhu	181	0.8341	0.9613	1.05
12	Huangqi–Baizhu	181	0.8341	0.9337	1.01
13	Taizishen–Baizhu and Fuling	174	0.8018	0.9253	1.07
14	Huangqi–Baizhu and Fuling	174	0.8018	0.9310	1.01
15	Taizishen–Baizhu and Huangqi	169	0.7788	0.9527	1.10
16	Fuling–Baizhu and Huangqi	169	0.7788	0.9586	1.05
17	Fuling–Baizhu and Taizishen	165	0.7604	0.9758	1.07
18	Huangqi–Baizhu and Taizishen	165	0.7604	0.9758	1.06
19	Fuling–Jineijin	163	0.7512	0.9141	1.00
20	Huangqi–Jineijin	163	0.7512	0.9080	0.99

### 3.3. Cluster analysis

**Figure 5.**
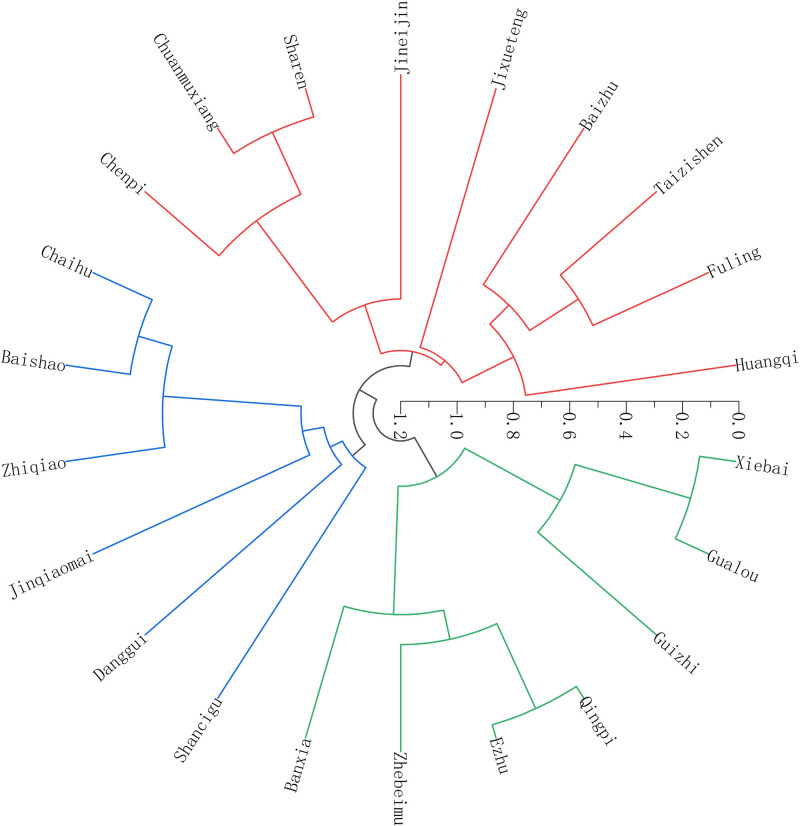
Hierarchical clustering of prescription groups.

Systematic clustering of high-frequency drugs was performed using IBM SPSS Statistics 27 and obtained a total of 3 core drug combinations (Table 3), followed by visualization of the drug cluster combinations using Origin 2024 (Fig. 5).

**Table 3:** The prescription composition of 3 clusters.

Sequence	Clustering combinations
1	Huangqi, Fuling, Taizishen, Baizhu, Jixuetang, Jineijin, Chuanmuxiang, Sharen, Chenpi
2	Chaihu, Baishao, Zhiqiao, Jinqiaomai, Danggui, Shancigu
3	Gualou, Xiebai, Guizhi, Ezhu, Qingpi, Banxia, Zhebeimu

### 3.4. Screening of drug active ingredients and disease targets

According to the above results, Huangqi, Fuling and Taizishen not only were the top 3 high-frequency medications, but comprised the combination with the highest association strength. Therefore, these 3 drugs could serve as a representation of medication laws of Professor Gang Xie to some extent. Hence, these 3 herbs were selected for subsequent network pharmacology analysis. After deduplicating and organizing the active ingredients of medicines obtained from the TCMSP and HERB databases, a total of 67 active ingredients were identified, including 36 from Huangqi, 17 from Fuling, and 16 from Taizishen. Then integrating data from the TCMSP and SwissTargetPrediction databases, a total of 770 ingredient-related targets were obtained.

**Figure 6.**
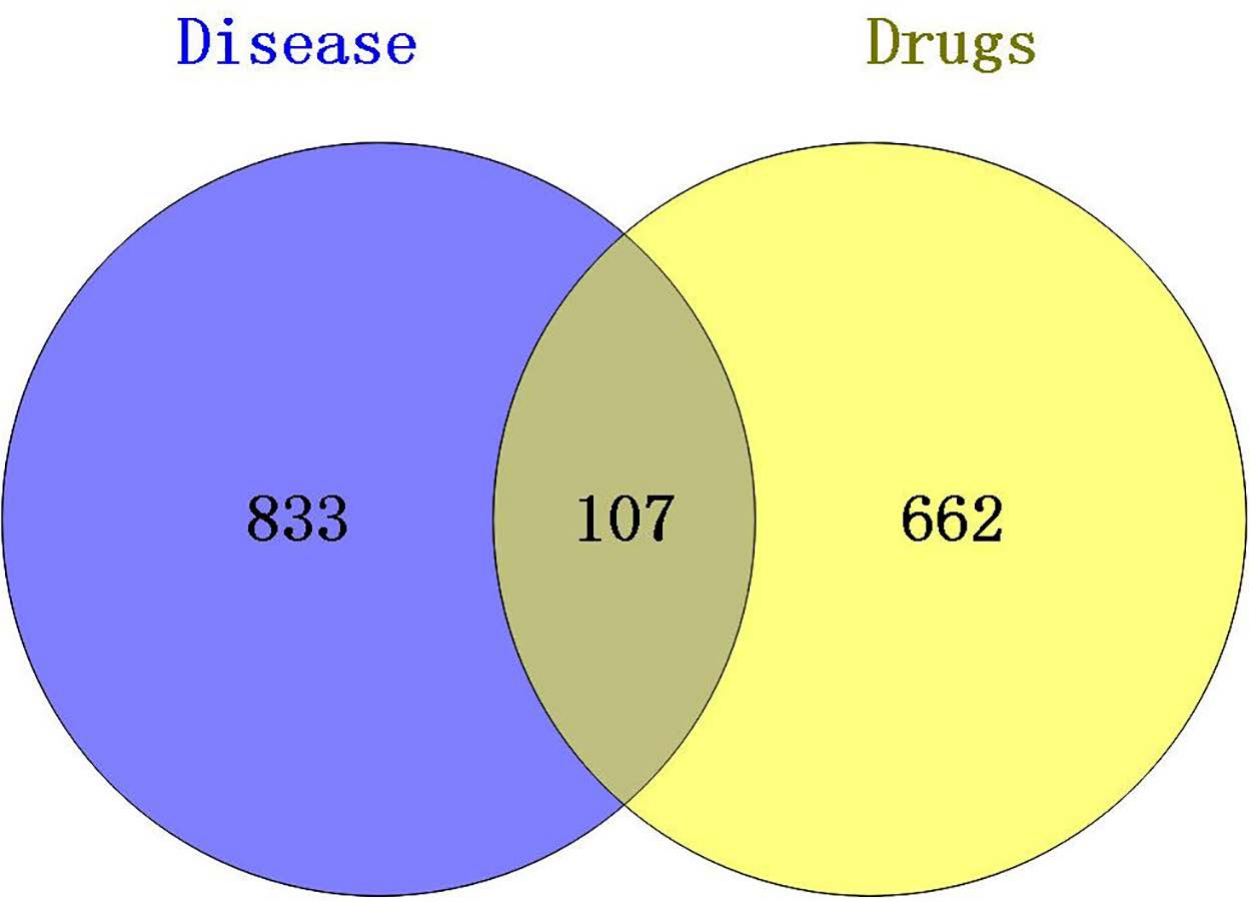
Intersecting targets of medicines and HFS. HFS = hand-foot syndrome after chemotherapy.

One high throughput sequencing data (GSE171468) was retrieved from the GEO database^[48]^ and 67 differential genes were screened by using GEO2R online tool. A total of 899 targets were obtained by searching the GeneCards and PharmGKB databases. After deduplication of these targets, 940 targets related to HFS were obtained. The intersection of the ingredient-related targets and the disease-related targets yielded 107 potential therapeutic targets (Fig. 6).

### 3.5. PPI network of the intersecting targets

**Figure 7.**
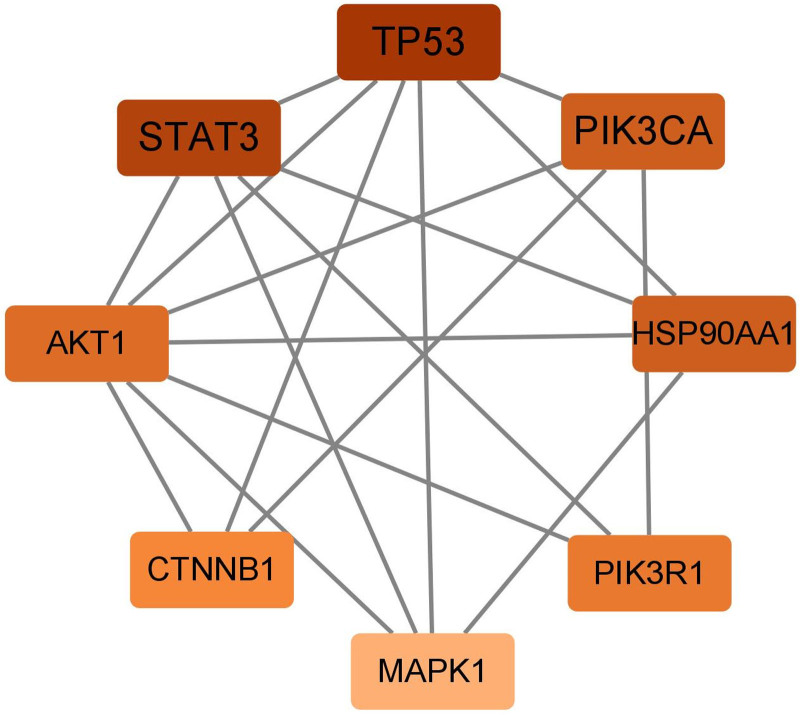
The core genes of intersecting targets.

107 potential targets of Huangqi, Fuling and Taizishen for the treatment of HFS were imported into the String platform and 107 nodes and 254 edges were obtained. The PPI network was further topologically analyzed by Cytoscape 3.8.0, and 8 core genes with the highest degree values were finally obtained (Fig. 7), including TP53, signal transducer and activator of transcription 3 (STAT3), phosphatidylinositol-3-kinase catalytic subunit alpha (PIK3CA), heat shock protein 90 alpha family class A member 1 (HSP90AA1), protein kinase B1 (AKT1), CTNNB1, phosphatidylinositol3-kinase regulatory subunit 1 (PI3KR1), and mitogen-activated protein kinase 1 (MAPK1).

### 3.6. GO and KEGG enrichment analysis

**Figure 8.**
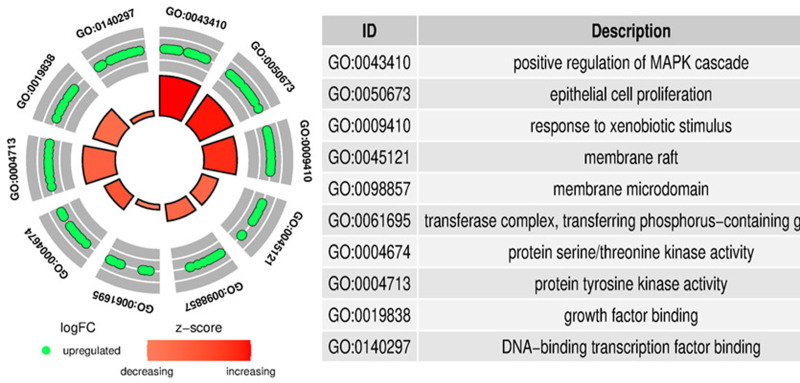
The top 10 items of GO enrichment analysis. GO = gene ontology.

**Figure 9.**
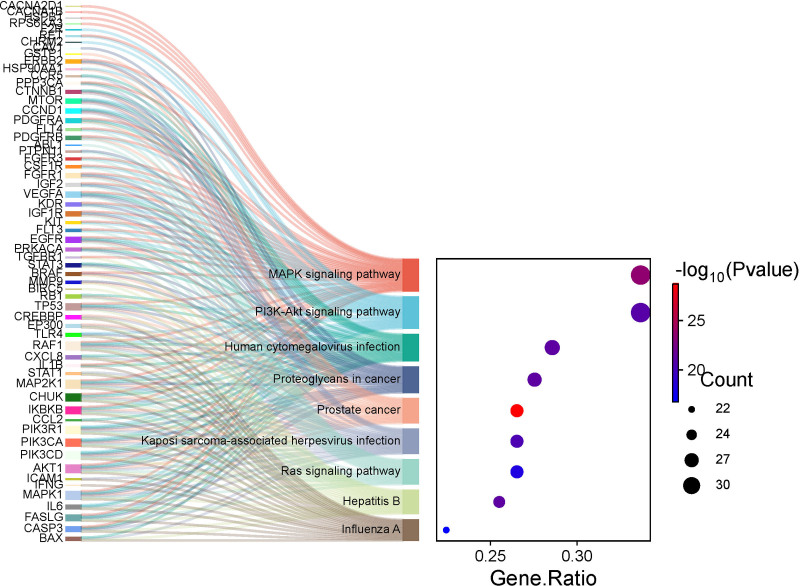
The top 10 items of KEGG pathways analysis. KEGG = Kyoto encyclopedia of genes and genomes.

GO and KEGG enrichment analysis of the intersecting targets were performed using R 4.1.1. GO analysis identified a total of 2411 biological processes, such as the positive regulation of MAPK cascade and epithelial cell proliferation; 48 cellular components, including membrane rafts and membrane microdomains; 140 molecular functions, such as protein serine/threonine kinase activity, protein tyrosine kinase activity and growth factor binding. KEGG pathway enrichment analysis revealed involvement in 174 signaling pathways, with key pathways including the MAPK signaling pathway, PI3K-Akt signaling pathway, proteoglycans in cancer and prostate cancer signaling pathway. The top 10 terms of both GO analysis (Fig. 8) and KEGG analysis (Fig. 9) were visualized using the Weishengxin platform.

### 3.7. Construction of “drug-ingredient-targets” network

**Figure 10.**
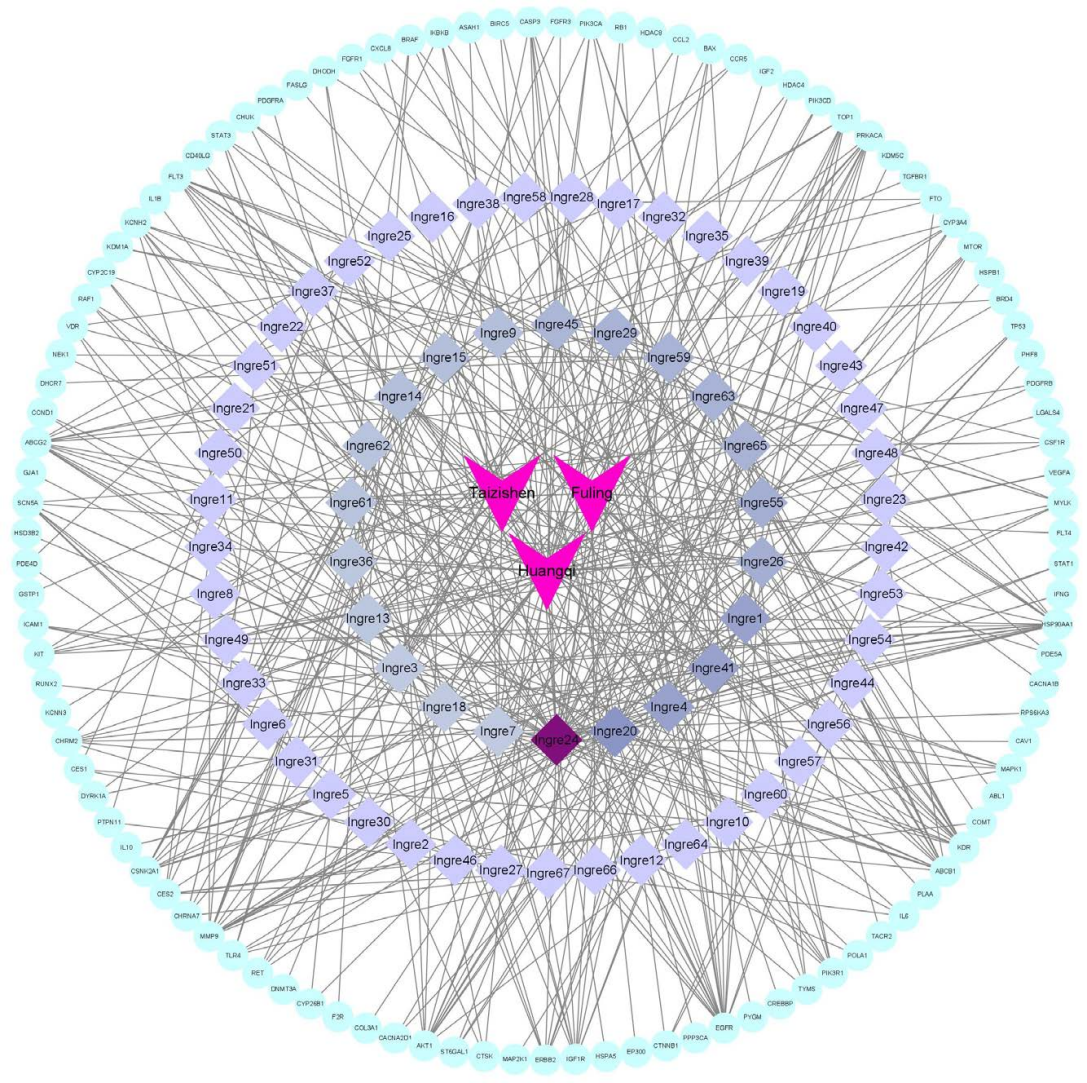
The “drug-ingredient-targets” network.

The active ingredients and potential therapeutic targets of Huangqi, Fuling and Taizishen were imported into Cytoscape 3.8.0 to construct the “drug-ingredient-targets” network (Fig. 10). Then the core active compounds were identified based on their degree values. The results indicated that quercetin, kaempferol, acacetin and luteolin exhibited the highest Degree values, suggesting that they were the key active ingredients of the “Huangqi–Fuling–Taizishen” combination in the treatment of HFS (Table 4).

**Table 4:** The key ingredients of the combination of Huangqi, Fuling and Taizishen.

Ingredient ID	Active ingredients	Degree values	Resources
Ingre24	Quercetin	44	Huangqi
Ingre20	Kaempferol	22	Huangqi
Ingre1	Acacetin	19	Taizishen
Ingre4	Luteolin	19	Taizishen

### 3.8. Molecular docking

**Figure 11.**
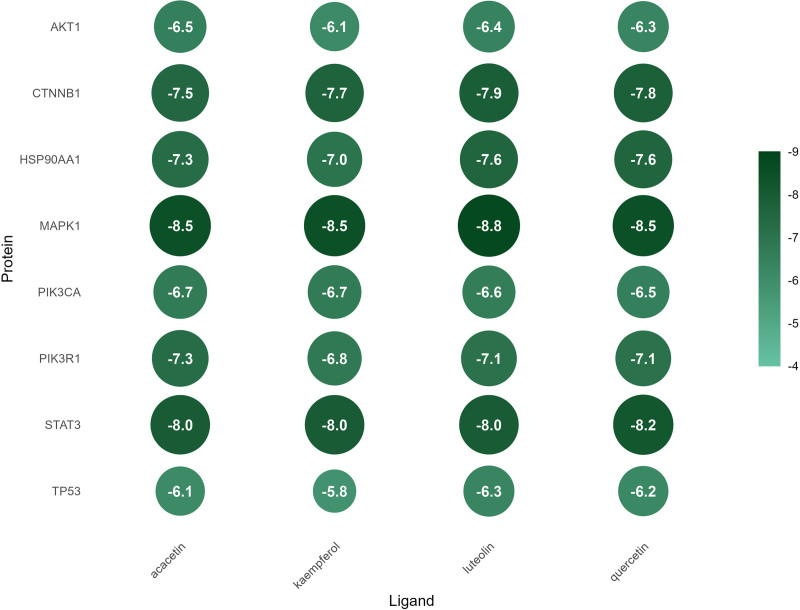
The docking energy between key active ingredients and core targets.

In this study, a total of 32 molecular docking intersections were performed between the 4 key active ingredients and 8 core targets. The results showed that quercetin, kaempferol, acacetin and luteolin exhibited a strong binding affinity with the core targets (Fig. 11).

## 4. Discussion

HFS is one of the common adverse effects after chemotherapy. The pathogenesis of HFS has not been fully clarified, modern medicine believes that it may be related to chemotherapeutics damaging the nerve myelin sheaths of distal extremities, mitochondrial damage and inflammation-induced axonal transport disorders.^[49–52]^ HFS has a high prevalence and it is difficult to recover, which can seriously affect the quality of daily life and reduce the efficacy of chemotherapy.^[53,54]^ In TCM theory, HFS can be categorized to “bi syndrome,” which is related to the imbalance of liver, spleen and kidney, mainly caused by the damage of chemotherapy drugs to middle jiao (spleen and stomach), resulting in qi and blood deficiency, as well as disharmony between the Ying-nutrients and Wei-defence.^[55]^ If the Wei-defence and Ying-nutrients were not full to nourish body, then the immunity would be weak. And the qi stagnation and blood stasis caused by chemotherapy also lead to qi and blood cannot reach the hands and feet by blocking the meridians, which will be disorder of the hands and feet.^[56]^

Professor Gang Xie summarized the pathogenesis of HFS as “deficiency of Ying and Wei with yi stagnation’. Based on the collected prescriptions, this paper found that the medication laws of Professor Xie in treating HFS predominantly involved the use of tonic medicines, phlegm-transforming medicines, and qi-regulating medicines. The nature of these herbs was mainly being warm, the tastes were mainly sweet and bitter, and they primarily targeted the liver, spleen and stomach meridians. Frequency statistics showed that drugs with the effect of tonifying qi and strengthening the spleen, such as Huangqi, Fuling and Taizishen, were the most frequently prescribed. These 3 herbs also formed the combination with the highest degree values in association analysis. “*Su Wen*” records that “Ying deficiency leads to numbness and Wei deficiency results in dysfunction, if both were deficient, numbness and dysfunction would occur simultaneously.” Huangqi, Fuling and Taizishen primarily act on the lung and spleen meridians to reinforce the organic function. The “*Bencao Qiuzhen*” said that Huangqi can “enter into lung to tonify the qi and strengthens the exterior to consolidate the Wei.” Modern pharmacological studies have shown that they can enhance immune function after chemotherapy and help alleviate neurodegenerative condition.^[57,58]^ In addition, other high-frequency herbs, such as Ezhu, Qingpi, Banxia, Chaihu, Zhiqiao and Baishao possess the effects of regulating qi and circulating blood. As recorded in “*Danxi Xinfa*,” “the numbness of the hands and feet is attributed to qi deficiency, dampness phlegm and stagnant blood. Numbness in all 10 fingers indicates the accumulation of dampness phlegm, and stagnant blood in the stomach.” These medication laws reflect a comprehensive consideration of qi deficiency, phlegm obstruction and blood stasis.

Through clustering analysis of high-frequency herbs, 3 groups of prescription formulas were ultimately identified. The cluster 1 comprised of Huangqi, Fuling, Taizishen, Baizhu, Jixueteng, Jineijin, Chuanmuxiang, Sharen, and Chenpi. This formula functions to tonify qi and strengthen the spleen and stomach. As recorded in “Su Wen,” “spleen governs the muscles of body.” This formula was similar to Sijunzi Decotion, a classic formula that tonify qi,^[59]^ and particularly suitable for patients who experience HFS related spleen–stomach deficiency syndrome. The cluster 2 consists of Chaihu, Baishao, Zhiqiao, Jinqiaomai, Danggui and Shancigu, while the cluster3 comprises Gualou, Xiebai, Guizhi, Ezhu, Qingpi, Banxia, and Zhebeimu. Their formulations are variants of Sini Powder and Sanleng Decotion and Gualou Xiebai Banxia Decotion respectively. These 2 clusters mainly aim to regulate qi, transform phlegm and circulate blood, which can provide useful ideas for HFS.

The results of network pharmacology analysis identified that quercetin, kaempferol, acacetin and luteolin were the key active ingredients of the core herbal combination “Huangqi–Fuling–Taizishen” in treating HFS. These flavonoids possess a wide range of pharmacological activities, including anti-tumor,^[60]^ anti-radiation^[61]^ and nephroprotective^[62]^ effects. Previous studies have shown that they can also exert synergistic effects with chemotherapeutic agents by enhancing efficacy and reducing toxicity, and they are capable of alleviating CIPN through multiple pathways.^[63–67]^ The mechanisms of these compounds may involve multiple approach to ameliorate the microenvironment of distal extremities. For example, quercetin has been reported to significantly improve capecitabine-induced HFS through targeting the thymidine phosphorylase in rats.^[68,69]^ Luteolin can improve peripheral nerve blood flow and conduction in rats by enhancing antioxidant activity through upregulating the expression of Nrf2 protein.^[70]^ Another research also proved that luteolin alleviate the doxorubicin-induced neurotoxicity by relieving inflammatory reaction.^[71]^ In addition, kaempferol also alleviated oxaliplatin-induced neurotoxicity by suppressing inflammation via the TLR4/NF-κB signaling pathway.^[72]^ Though evidence for acacetin, a flavonoid with an extraordinary potency in tumor treatment and reduce the chemotherapy-induced complications,^[73–75]^ in treating HFS is still absent, results in this article preliminary revealed its therapeutic potential. Therefore, results highlight the imperative for further investigating the therapeutic effect of acacetin in HFS management.

TP53, STAT3, PIK3CA, HSP90AA1, AKT1, CTNNB1, PIK3R1 and MAPK1 were screened as the core targets of the Huangqi–Fuling–Taizishen combination against HFS. These targets participated in the pathogenesis of HFS through various ways. PIK3CA, which encodes PI3K, has been well documented in cancer progression and chemoresistance together with AKT1.^[76–78]^ And the accumulation of paclitaxel has been shown to aggravate the peripheral neuropathy through PI3K-Akt pathway.^[79,80]^ MAPK1 and STAT3 were the key proteins in the process of intracellular signaling and regulated cell growth and inflammatory responses, the 2 proteins both promote capecitabine-associated HFS by upregulating COX-2, IL-6, and IL-8.^[81]^ TP53, a downstream effector of both PI3K-AKT and MAPK pathways critical for cell cycle regulation. Study has shown that it can trigger apoptosis and DNA damage in normal skin cells when activated by chemotherapeutic agents, thereby inducing HFS.^[82]^ Mutations in CTNNB1 can lead to progress in multiple tumors.^[83,84]^ A recent study demonstrated that the upregulation of CTNNB1 protein promoted the development of CIPN.^[85]^ Additionally, HSP90AA1 was implicated in tumor development and chemoresistance,^[86,87]^ as well as the process of cisplatin-induced CIPN.^[88]^ KEGG enrichment analysis revealed that these intersecting targets were predominantly involved in the PI3K-Akt and MAPK pathways. The PI3K-Akt pathway enhances cell survival and inflammation through AKT1 phosphorylation. Research has demonstrated that the inhibition of PI3K-Akt pathway can alleviate vincristine-induced CIPN, suggesting that the potential of PI3K-Akt in treating HFS.^[89]^ Moreover, the key role of MAPK pathway in the pathogenesis of CIPN have been clarified in many researches, it can promote the development of CIPN through activating oxidative stress and downstream inflammatory signal factors.^[90,91]^ Moreover, molecular docking showed strong binding affinities between the key active compounds and core targets, suggesting medicines may alleviate HFS through multiple pathways and targets.

Although the valuable insights provided by this study, several limitations should be acknowledged. First, all prescriptions were derived from the clinical practice of a single senior TCM oncologist, which may introduce selection bias. The prescribing patterns may therefore partly reflect individual clinical preferences rather than universally representative TCM practice. Second, the analysis relied primarily on data mining and network pharmacology approaches, and the mechanistic results remain at the in-silico prediction stage. Experimental validation through cells or animal models will be required to substantiate these results. And after that, it is necessary to develop clinical trials to validate the therapeutic effect. Third, as a retrospective study based on outpatient medical records, detailed clinical parameters such as symptom grading and severity differences could not be comprehensively assessed. In fact, most HFS patients come to see a doctor for abnormal sensation. Therefore, in this study, we focus on the improvement of sensory abnormalities (such as numbness and pain), which is usually considered to be part of the manifestations of HFS. In addition, the clustering results may have been influenced by patient composition. For example, Gualou Xiebai Banxia Decoction was frequently used in Professor Xie’s practice for lung cancer patients. Since lung cancer accounts for a high proportion of cases in the outpatient population, and some chemotherapeutic agents (such as docetaxel) commonly used for this disease are strongly associated with a higher incidence of HFS, the clustering outcome may partly reflect disease-related prescribing bias. These limitations notwithstanding, the study provides exploratory evidence and establishes a foundation for future multicenter, prospective, and experimental research to further clarify the molecular basis of TCM prescribing patterns in chemotherapy-induced HFS.

## 5. Conclusion

This study employed data mining and network pharmacology technology to explore the medication laws of Professor Gang Xie in treating HFS. Professor Xie’s therapeutic strategies were based on qi deficiency, qi stagnation, phlegm–dampness and blood stasis, with treatment principles focused on tonifying qi, strengthening spleen, regulating qi and circulating blood. The herbs commonly used in prescriptions included Huangqi, Fuling, Taizishen, Chaihu, Zhiqiao, Ezhu, Qingpi, and Banxia. And the natures of these herbs were common warm, flavors were sweet and bitter, as well as the meridians were spleen, stomach and liver. Network pharmacology analysis of the core combination of Huangqi–Fuling–Taizishen identified quercetin, kaempferol, acacetin and luteolin as the key active compounds may act on HFS through targets such as TP53, STAT3 and PIK3CA and via the PI3K-Akt and MAPK pathways. Drawing on the reliable outpatient case records, this work offers a valuable perspective for future treatment of HFS. Nonetheless, because of the inherent limitation of data mining and network pharmacology, future experiments should be conducted to validate these mechanisms in vitro and in vivo, and the therapeutic effect should be further evaluated in clinical trials.

## Acknowledgments

Thanks to Yanmei Wang, Luchuan Yang and Yuanming Liu’s support to this study.

## Author contributions

**Funding acquisition:** Gang Xie.

**Investigation:** Li Deng.

**Methodology:** Li Deng.

**Project administration:** Gang Xie.

**Software:** Li Deng, Hongjing Chen.

**Visualization:** Li Deng, Hongjing Chen.

**Writing – original draft:** Li Deng, Hongjing Chen.

**Writing – review & editing:** Li Deng.

## Correction

This article was originally published without the funding source details. The funding information has now been included in the online version as a first page footnote.
